# Moxidectin steady state prior to inoculation protects cats from subsequent, repeated infection with *Dirofilaria immitis*

**DOI:** 10.1186/s13071-015-0710-z

**Published:** 2015-02-18

**Authors:** Susan E Little, Joe A Hostetler, Jennifer E Thomas, Keith L Bailey, Anne W Barrett, Kaylynn Gruntmeir, Jeff Gruntmeir, Lindsay A Starkey, Chris Basel, Byron L Blagburn

**Affiliations:** Department of Veterinary Pathobiology, Center for Veterinary Health Sciences, Oklahoma State University, Stillwater, OK USA; Bayer HealthCare, Animal Health, Shawnee, KS USA; Oklahoma Animal Disease Diagnostic Laboratory, Center for Veterinary Health Sciences, Oklahoma State University, Stillwater, OK USA; Department of Veterinary Pathobiology, College of Veterinary Medicine, Auburn University, Auburn, AL USA

**Keywords:** Cat, *Dirofilaria immitis*, Heartworm, Moxidectin, Steady state

## Abstract

**Background:**

Infection of cats with *Dirofilaria immitis* causes seroconversion on antibody tests and pulmonary pathology, often without subsequent development of adult heartworms. Consistent administration of topical 10% imidacloprid-1% moxidectin has been shown to result in sustained plasma levels of moxidectin in cats after three to five treatments, a pharmacokinetic behavior known as “steady state”.

**Methods:**

To evaluate the ability of moxidectin at “steady state” to protect cats from subsequent infection with *D. immitis*, cats (n = 10) were treated with the labeled dose of topical 10% imidacloprid-1% moxidectin for four monthly treatments. Each cat was inoculated with 25 third-stage larvae of *D. immitis* 7, 14, 21, and 28 days after the last treatment; non-treated cats (n = 9) were inoculated on the same days, serving as infection controls. Blood samples were collected from each cat from 1 month prior to treatment until 7 months after the final inoculation and tested for antibody to, and antigen and microfilaria of, *D. immitis*.

**Results:**

Measurement of serum levels of moxidectin confirmed steady state in treated cats. Cats treated with topical 10% imidacloprid-1% moxidectin prior to trickle inoculation of *D. immitis* L3 larvae throughout the 28 day post-treatment period remained negative on antibody and antigen tests throughout the study and did not develop gross or histologic lesions characteristic of heartworm infection. A majority of non-treated cats tested antibody positive by 3–4 months post infection (6/9) and, after heat treatment, tested antigen positive by 6–7 months post-infection (5/9). Histologic lesions characteristic of *D. immitis* infection, including intimal and medial thickening of the pulmonary artery, were present in every cat with *D. immitis* antibodies (6/6), although adult *D. immitis* were confirmed in only 5/6 antibody-positive cats at necropsy. Microfilariae were not detected at any time.

**Conclusions:**

Taken together, these data indicate that prior treatment with 10% imidacloprid-1% moxidectin protected cats from subsequent infection with *D. immitis* for 28 days, preventing both formation of a detectable antibody response and development of pulmonary lesions by either immature stages of *D. immitis* or young adult heartworms.

## Background

Infection with heartworm (*Dirofiliaria immitis*) can induce a severe, potentially fatal disease in cats that in some patients manifests as respiratory distress or sudden death [1]. In areas of the southern United States where ample infected dog reservoir hosts are found, necropsy surveys document that as many as 16% of cats may be infected with adult *D. immitis,* although there is a much lower reported rate of detection using commercial antigen tests [2,3]. However, antibody testing, which reveals evidence of both past and current infection, documents a higher prevalence than antigen tests, particularly in cats with respiratory disease. This suggests many cats develop aborted infections, presumably due to the robust immune response provoked by migrating, developing larvae [4]. Although short-lived, these aborted infections have been shown to result in pulmonary pathology [5].

Four different macrocyclic lactones are label approved, either alone or in combination with other active ingredients, to protect cats from infection with adult *D. immitis* in the United States: ivermectin and selamectin, which are avermectins, and moxidectin and milbemycin oxime, which are milbemycins [6]. The moxidectin-based feline heartworm preventive is applied topically then absorbed systemically, and is formulated in combination with imidacloprid, an insecticide primarily intended to control flea infestations [7]. Each of these heartworm preventives was approved based on the WAAVP-endorsed experimental approach whereby infection is first established by inoculation with third-stage larvae and then 30 days later the preventive is administered [8]. The number of adult worms that develop in treated animals is compared to that in non-treated controls to determine efficacy [9]. Because infections are allowed to progress for one month using this approach, seroconversion on antibody tests may occur even if adult heartworms do not develop in treated cats.

Consistent administration of topical 10% imidacloprid-1% moxidectin has been shown to result in sustained, elevated plasma levels of moxidectin in treated cats, a phenomenon referred to as “steady state”; the same phenomenon is documented to occur with the canine product (10% imidacloprid-2.5% moxidectin) [9]. To determine if steady state would protect cats from *D. immitis* infection prospectively for 28 days, and thus prevent development of successful infection and a detectable antibody response, cats were treated with four consecutive monthly treatments of 10% imidacloprid-1% moxidectin and then, after steady state was achieved, inoculated weekly for four weeks with third-stage larvae of *D. immitis* to monitor the serologic and pathologic results of infection.

## Methods

### Animals

Twenty 6- to 8-month-old male and female intact domestic short haired cats purchased from a commercial supplier were used in this study. Throughout the study cats were individually housed in climate controlled indoor facilities. To ensure lung pathology was not confounded by non-study materials, silica-free litter was used through the study (corn cob bedding, Harlan, Indianapolis, IN). Prior to enrollment in the study, physical examinations were conducted and all cats were found to be negative on commercial test for heartworm antigen, feline leukemia antigen, and feline immunodeficiency virus antibody (SNAP®Feline Triple® Test, IDEXX Laboratories, Westbrook, Maine). Cats were stratified by body weight and randomly allocated into two groups, treatment (n = 10) or control (n = 10). One control cat was removed from the study prior to infection due to detection of a pre-existing cardiac condition; necropsy revealed hypertrophic cardiomyopathy. The 19 remaining cats (10 treatment, 9 control) were housed in adjacent animal rooms in Oklahoma State University’s (OSU) Association for Assessment and Accreditation of Laboratory Animal Care (AAALAC)-accredited laboratory animal facilities and cared for by Laboratory Animal Resources staff following standard care protocols throughout the experiment. All animal care and use protocols were approved by the OSU Institutional Animal Care and Use Committee prior to initiation of the research.

### Treatment and assessment of steady-state

Topical 10% imidacloprid-1% moxidectin (AdvantageMulti® for Cats, Bayer HealthCare LLC, Animal Health Division) was administered according to label directions on study days −84, −56, −28, and 0; control cats were not treated. To monitor moxidectin levels before and during challenge infection with *D. immitis*, whole blood was collected from each cat on study days −85, −57, −29, −1, 7, 14, 21, and 28, placed in vacutainer tubes without anticoagulant, centrifuged, the serum harvested, and aliquots stored at -80C. Once all samples had been collected and frozen, serum samples were shipped on dry ice to a reference laboratory (Bayer HealthCare, Monheim, Germany) for determination of moxidectin levels by mass spectroscopy. Briefly, serum samples for pharmacokinetic evaluation were analyzed using a validated HPLC-MS/MS method for the determination of moxidectin and ivermectin in serum (Bayer CropScience AG Method 01086/M001). Samples were first deproteinized by mixing 200 μL of serum with 800 μL of a precipitation solution and then filtered. The precipitation solution was prepared by mixing 100 mL of a solution of 0.39 g ammonium acetate in 1 L water with 1 mL of formic acid and 600 mL of acetonitrile. The quantitative determination was performed by HPLC with tandem mass spectrometric detection using an AB Sciex API 4000 mass spectrometer. The lower limit of quantitation was 1.0 μg/L.

### Inoculation with ***Dirofilaria immitis***

Third-stage larvae of *D. immitis* (Missouri strain) were harvested from *Aedes aegypti* mosquitoes infected by artificially feeding on microfilaremic blood as previously described [10]. Larvae (n = 25) were subcutaneously inoculated in the inguinal region of each cat on study days 7, 14, 21, and 28.

### Heartworm tests

Whole blood samples were collected from the jugular vein of each cat into vacuum tubes containing EDTA and tubes with no anticoagulant on study days 56, 84, 112, 140, 168, 196, and 224. Knott tests were performed on fresh EDTA anti-coagulated whole blood samples collected on days 196 and 224 as previously described [11]. To detect commensal *Wolbachia* spp. bacteria associated with *D. immitis*, total nucleic acid was extracted from frozen (−20°C) EDTA anti-coagulated whole blood samples collected on days 168, 196, and 224 and real time PCR for *Wolbachia* spp. performed as previously described [12]. Serum was harvested by centrifugation and stored at −80°C until tested for *D. immitis* antigen (days 140, 168, 196, and 224) and antibody (days 56, 84, 112, 140, 168, 196, and 224). Antigen tests were performed using commercial microtiter well assays (DiroCHEK®, Zoetis, Florham Park, New Jersey) according to manufacturer’s instructions for laboratory assays. Antibody tests were performed in triplicate by a reference laboratory (ANTECH Diagnostics, Irvine, California) according to standard laboratory protocols with optical density reported.

### Post-mortem examination

On study day 224, cats were euthanized by barbiturate overdose, the abdominal and thoracic cavity briefly examined for aberrant *D. immitis*, and the heart and lungs removed. Airways were gently infused with formalin after collection to preserve histologic architecture. The heart, main pulmonary arteries, and associated branches were dissected and all *D. immitis* present were collected and placed in fixative. Sections of the pulmonic trunk, kidney, and oblique sections of the medial and lateral aspect of the right caudal lung lobe were placed in 10% neutral buffered formalin and processed for histopathologic examination as previously described [5]. Paraffin-embedded sections were stained with hematoxylin and eosin and examined by the same ACVP-board certified pathologist masked to treatment group. Lesions present in lung, pulmonic trunk, and kidney were graded for severity (0 = none, 1 = minimal, 2 = mild, 3 = moderate, 4 = severe).

#### Statistics

The number of heartworms recovered at necropsy from treated and non-treated control cats, and the difference in pathology scores for cats in each group were compared using a Wilcoxon’s Rank Sum Test [13]. An alpha value of 0.05 was assumed and statistical computations made using SAS® 9.2 Macro Language (SAS Institute Inc., Cary, North Carolina).

## Results

**Figure 1:**
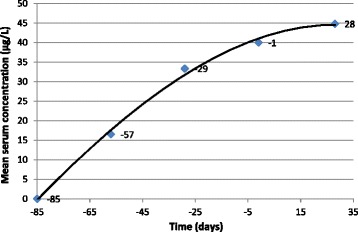
**Mean levels of moxidectin (μg/L) prior to and during repeated infection with** ***Dirofilaria immitis*** **.** Cats were treated with 10% imidacloprid-1% moxidectin on study days −84, −56, −28, and 0. Third-stage larvae of *D. immitis* (n = 25) were inoculated on days 7, 14, 21, and 28, 1 – 4 weeks after the final treatment was administered.

Mean trough levels of moxidectin in samples collected from treated cats on study days −85, −57, −29, and −1, prior to first inoculation with *D. immitis*, were as follows: none detected, 16.5, 33.4, and 40.0 μg/L, respectively (Figure 1). Mean levels of moxidectin during the repeated inoculations with *D. immitis* at study days 7, 14, 21, and 28 were as follows: 65.2, 53.6, 49.0, and 44.8 μg/L, respectively. Moxidectin was not detected in samples from non-treated control cats at any time point.

Antigen was detected in serum of 0, 0, 1, and 1 of the non-treated cats at study day 140, 168, 196, and 224, respectively. As previously reported, heat treatment of sera from the non-treated cats revealed antigen in 1, 5, and 5 cats at study days 168, 196, and 224, respectively [14]. In evaluation at the commercial reference lab, antibody was detected in 0, 5, 6, 6, 6, 6, and 6 of the non-treated cats at study day 56, 84, 112, 140, 168, 196, and 224. Antigen (before and after heat treatment) and antibody test results on 6 of the non-treated cats were reported in an earlier publication focusing on immune complexes in cats infected with *D. immitis* [14]. All cats in the treated group remained antibody and antigen negative at all time points.

Adult heartworms were found at necropsy in 5/9 non-treated cats, including 5/6 antibody-positive non-treated cats; 1, 1, 2, 2, or 6 worms were recovered from each infected cat, respectively. No worms were identified in any treated cat. Although three control cats did not become infected, non-treated control cats were significantly more likely to be infected with heartworms than treated cats (*P* = 0.02). Grossly, the lungs from infected cats appeared diffusely mottled and edematous with moderate to marked rugose thickening of the intimal layer of the main pulmonary artery and branches. Examination of lung tissue from a sixth non-treated cat, which was also antibody and, after heat treatment, antigen positive, revealed gross lesions consistent with *D. immitis* infection (e.g. pulmonary thromboembolism) but intact nematodes or nematode fragments were not recovered. Nematodes and gross lesions were present in 0/10 cats inoculated after moxidectin steady state was achieved.

**Figure 2:**
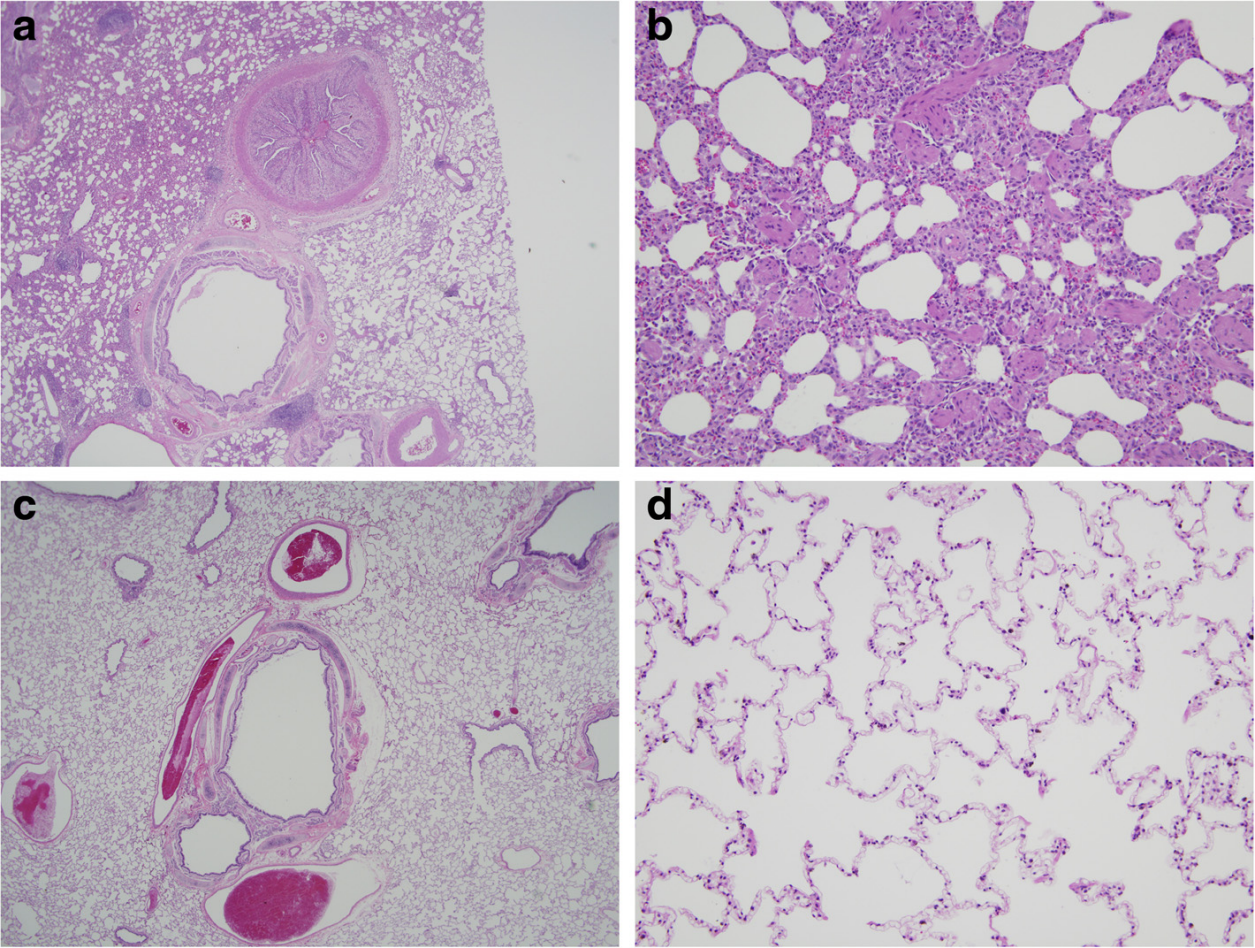
**Photomicrograph of hematoxylin & eosin-stained sections of lung. (a)** Intimal thickening, villous proliferation and leukocyte infiltration of the pulmonary artery (arrow) associated with *Dirofilaria immitis* infection in a non-treated control cat (10× magnification). **(b)** Medial thickening of pulmonary artery branches (arrowheads) associated with *Dirofilaria immitis* infection in a non-treated control cat (20× magnification). **(c, d)** In cats treated with 10% imidacloprid-1% moxidectin prior to repeated inoculation with *D. immitis*, pulmonary lesions did not develop (10× and 20× magnification, respectively).

Microscopic examination of lung tissue of non-treated cats revealed significant thickening and villous proliferation of the intimal layer of the pulmonary artery (6/9 cats; mean severity score = 3.7; *P* = 0.133), leukocyte infiltration of the intimal layer of pulmonary artery (6/9 cats; mean severity score = 3.2; *P* = 0.133), and medial thickening of the pulmonary artery (5/9 cats; mean severity score = 2.3; *P* = 0.434). Non-treated cats also had intimal thickening (4/9 cats; mean severity score = 1.7) and intimal and adventitial leukocyte infiltration (4/9 cats; mean severity score = 1.8) evident in the pulmonic trunk, although the assigned scores were not significantly different than those in the treated cats (*P* = 0.113). Similar lesions were not present in treated cats (Table 1, Figure 2). No significant lesions were present in the kidney of any cats in this study. Numbers of nematodes, antigen test results, antibody test results, and significant histopathologic lesions consistent with *D. immitis* infection according to cat number are provided in Table 1.

**Table 1:** **Numbers of** ***Dirofilaria immitis*** **recovered at necropsy, antigen test results, antibody test results, and significant histopathologic lesions consistent with heartworm infection in cats inoculated with** ***D. immitis***

			**Pulmonary artery lesion score**			**Pulmonic trunk lesion score**	
**Non-treated cats (No.** ***D. immitis*** **recovered)**	Antigen*	Antibody*	**Intima, thickening and villous proliferation**	**Intima, leukocyte infiltration**	**Media, thickening**	**Intima, thickening**	**Intima and adventitia, leukocyte infiltration**
1 (0)**	NEG	**POS**	**4**	**3**	**4**	**2**	**3**
2 (6)	**POS**	**POS**	**4**	**3**	**1**	**2**	**2**
3 (1)	NEG	**POS**	**4**	**4**	**4**	**4**	**3**
4 (2)	NEG	**POS**	**2**	**2**	0	0	0
5 (1)	NEG	**POS**	**4**	**4**	**3**	0	0
6 (2)	NEG	**POS**	**4**	**3**	**2**	**2**	**3**
7 (0)	NEG	NEG	0	0	0	0	0
8 (0)	NEG	NEG	0	0	0	0	0
9 (0)	NEG	NEG	0	0	0	0	0
Treated							
11 (0)	NEG	NEG	0	0	0	0	0
12 (0)	NEG	NEG	0	0	0	0	0
13 (0)	NEG	NEG	0	0	0	0	0
14 (0)	NEG	NEG	0	0	0	0	0
15 (0)	NEG	NEG	0	0	0	0	0
16 (0)	NEG	NEG	0	0	0	0	0
17 (0)	NEG	NEG	0	1	0	0	0
18 (0)	NEG	NEG	0	0	0	0	0
19 (0)	NEG	NEG	0	0	0	0	0
20 (0)	NEG	NEG	0	0	0	0	0

*Antigen and antibody results from cats 1–6 were published previously; 5/6 cats converted to antigen positive following heat treatment of serum to disrupt immune complexes [13]. None of the non-infected cats (cats 7–9, 11–20) converted to antigen positive with heat treatment (data not shown). **No worms were recovered but severe gross and histologic pulmonary lesions were present.

Treated cats received four monthly topical treatments with 10% imidacloprid-1% moxidectin prior to, but not after, inoculations; data provided were collected on study day 224. (Positive or significant results in bold).

## Discussion

Traditionally, monthly heartworm preventives have been used to prevent heartworm disease by administering them to pets after infection has already occurred. For this reason, preventives are largely tested for efficacy one month after infection with third-stage larvae [15]. However, topical moxidectin is unusual in that it has unique pharmacokinetic properties compared to other macrocyclic latones, resulting in a much longer mean residence time after treatment (Figure 1) [6]. The data in the present paper demonstrate that prior treatment with topical 10% imidacloprid-1% moxidectin protected cats from subsequent infection with *D. immitis* for as long as 4 weeks after the last preventive dose was administered, without further treatment, blocking both formation of a detectable antibody and antigen response and development of *D. immitis*-induced pulmonary lesions. The steady-state levels of moxidectin achieved by prior treatment (Figure 1) appear to have eliminated *D. immitis* larvae upon or shortly after each repeated inoculation, abrogating infections and preventing pulmonary damage.

The severe pulmonary lesions evident in the non-treated, antibody-positive cats in the present study were similar to those described previously from both experimental and spontaneous feline *D. immitis* infection [5,16-18]. Clinical signs were not observed in any of the cats during this study although disease due to *D. immitis* has been documented in the literature. Sudden death can occur, and in one prospective study, the majority of cats naturally infected with *D. immitis* developed clinical signs (53.5%) or died (20.9%) from the infection [1,19]. The gross and microscopic lesions in infected cats in the present study are consistent with heartworm-associated lung damage; had infections been allowed to proceed, some of the non-treated cats may have gone on to develop clinical signs [1].

Cats treated with 10% imidacloprid-1% moxidectin prior to inoculation also did not develop a positive antibody response as determined by a commonly used referral laboratory. Antibody tests in cats may become positive as early as 2 months after infection, although levels appear to wane thereafter [20,21]. In the present study, positive antibody test results were only obtained on samples collected from non-treated cats with gross or microscopic lesions associated with *D. immitis* infection, but not in three non-treated cats that apparently did not become infected or in any of the treated cats (Table 1). In experimental heartworm infections, approximately 75% of inoculated cats become infected (AHS, 2014), a percentage similar to that observed in the present study (6/9, 66.7%). Most likely, infections failed to establish in three of the non-treated cats; moxidectin was not detected in serum from any of the non-treated cats at any time, and all control cats were housed separately from the treated cats throughout the study. However, all of the cats that received moxidectin were protected from successful heartworm infection even though treatment ceased prior to the repeated inoculations. Steady-state levels of moxidectin remaining from the previous treatments appear to have protected them from subsequent infection in a manner similar to that described for hookworms [9].

Interestingly, detection of antigen of *D. immitis* was rare in this study, even in non-treated cats from which adult heartworms were collected at necropsy (Table 1). Heat treatment of serum samples from these cats was previously reported to disrupt immune complexes, enhancing antigen detection [14]. A serum sample from one cat in this study that was initially antigen negative became positive after heat treatment [14]. This cat was antigen positive without heating at study day 196, antibody positive from day 112–224, and had clear histopathologic lesions of *D. immitis* infection even though intact nematodes were not recovered at necropsy (Table 1, Cat 1). Antigen may have been detected from heartworms that died, presumably due to immune clearance, prior to necropsy examination. Elevated levels of antibody, particularly early in infection or as worms die, can result in formation of antigen-antibody complexes which prevent detection of antigen [22]. Alternatively, this cat may have had an ectopic infection which was overlooked on necropsy examination; ectopic migration of *D. immitis* to the abdominal and pleural cavities is common in cats [1].

The lack of antibody seroconversion in moxidectin treated cats observed in the present study suggests larvae were killed shortly after inoculation by the presence of existing, steady state levels of moxidectin; early death of larvae also protected the treated cats from subsequent pulmonary pathology (Table 1). Topical moxidectin is unusual among the macrocyclic lactones in that it has unique pharmacokinetic properties and remains at detectable levels for weeks after administration [6]; this phenomenon was confirmed by measuring serum levels in the present study (Figure 1). Other monthly macrocyclic lactone-based heartworm preventives are not known to result in sustained levels of active between treatments. Similar studies to investigate protection of cats from seroconversion or pathology have been conducted with selamectin and ivermectin [23-26]. Cats treated with ivermectin after infection become antibody positive, although the titer is generally lower than that of untreated cats [23]. Another study showed that infection followed by treatment 30 days later with selamectin resulted in lower pulmonary lesion scores than non-infected cats, although cats receiving selamectin became antibody positive [25,26]. A more recent study documented that pretreatment with selamectin one month and again 2 days before single infection with *D. immitis* larvae eliminated pathology and seroconversion when monthly treatments continued to be consistently administered throughout the entire infection period [27]. In the present study, topical 10% imidacloprid-1% moxidectin treatments were only administered prior to infection; nonetheless, the presence of moxidectin steady-state apparently still interrupted infection, preventing both pathology and seroconversion even in the face of repeated inoculation with *D. immitis*.

## Conclusions

Previous work has shown that, like other monthly heartworm preventives, topical 10% imidacloprid-1% moxidectin eliminates *D. immitis* larvae acquired in the past month [7]. The present study provides evidence that this duration of protection also continues forward, after administration of the preventive ceases, and that this protection into the month following the last administration protects cats from antibody seroconversion, successful infection with *D. immitis*, and associated pulmonary pathology.
